# A comparability study of 5 commercial *KRAS *tests

**DOI:** 10.1186/1746-1596-5-23

**Published:** 2010-04-16

**Authors:** Kelly Oliner, Todd Juan, Sid Suggs, Michael Wolf, Ildiko Sarosi, Daniel J Freeman, Tibor Gyuris, Will Baron, Andreas Bakker, Alex Parker, Scott D Patterson

**Affiliations:** 1 Department of Molecular Sciences, Amgen Inc., Thousand Oaks, CA, USA; 2 Department of Protein Sciences, Amgen Inc., Thousand Oaks, CA, USA; 3 Department of Global Biostatistics & Epidemiology, Amgen Inc., Thousand Oaks, CA, USA; 4 Department of Clinical Development, Amgen Inc., Thousand Oaks, CA, USA; 5 Department of Pathology, Amgen Inc., Thousand Oaks, CA, USA; 6 Department of Oncology Research, Amgen Inc., Thousand Oaks, CA, USA; 7 Department of Global Technical Services, Amgen Inc., Thousand Oaks, CA, USA; 8 Department of Molecular Sciences, Amgen Inc., Cambridge, MA, USA

## Abstract

**Background:**

Activating mutations in the *KRAS *gene occur frequently in human tumors, including colorectal carcinomas; most mutations occur in codons 12 and 13. Mutations in *KRAS *have been associated with poor response to anti-epidermal growth factor receptor antibodies. Therefore, an accurate and readily available analysis of *KRAS *mutational status is needed. The aim of this study was to evaluate concordance between *KRAS *assays performed by 6 different laboratories.

**Methods:**

Forty formalin-fixed paraffin-embedded colorectal cancer tumor samples were obtained. Sample sections were submitted for *KRAS *mutation analysis to 5 independent commercial laboratories (Agencourt, Gentris, Genzyme, HistoGeneX, and Invitek) and to the Amgen DNA Sequencing Laboratory for direct polymerase chain reaction sequencing. The assay used by Invitek is no longer commercially available and has been replaced by an alternative technique. Results from the commercial services were compared with those from Amgen direct sequencing by κ statistics.

**Results:**

*KRAS *mutations were observed in codon 12 and/or 13 in 20 of 40 (50%) samples in Amgen direct sequencing assays. Results from HistoGeneX (κ = 0.95), Genzyme (κ = 0.94), and Agencourt (κ = 0.94) were in almost perfect agreement with these results, and the results from Gentris were in substantial agreement with the results from Amgen (κ = 0.75). The Invitek allele-specific assay demonstrated slight agreement (κ = 0.13).

**Conclusions:**

This study provides data on the comparability of *KRAS *mutational analyses. The results suggest that most (but not all) commercial services provide analysis that is accurate and comparable with direct sequencing.

## Background

Inhibitors of epidermal growth factor receptor (EGFR), including the monoclonal antibodies panitumumab and cetuximab, have recently emerged as treatment options for metastatic colorectal cancer (mCRC) [1,2]. Mutations in *KRAS *have been associated with poor responses to both cetuximab and panitumumab in patients with CRC [3]. The aim of this study was to evaluate comparability among *KRAS *assays performed by 6 different laboratories in order to identify a vendor and assay that would be used to determine the clinical utility of *KRAS *in our pivotal panitumumab trial in mCRC [4].

## Methods

### Tissue Samples and DNA Isolation

Formalin-fixed paraffin-embedded (FFPE) human CRC samples (N = 40) were obtained from the following procurement service providers: Asterand plc (Detroit, MI), Ardais Corp (Lexington, MA), and the National Disease Research Interchange (Philadelphia, PA). Fourteen (35%) samples were from men, 20 (50%) were from women, and 6 (15%) were unassigned (Table 1). The median age was 67 years (range, 35-94 y); age data were not available for 7 patients. The samples were primary resections from colon adenocarcinoma (n = 36), rectum adenocarcinoma (n = 3), and rectum carcinoma (n = 1) with a range of poorly to well-differentiated tumors of different stages with variable tumor, normal, stromal, and necrotic content. The aim was to select samples that were representative of samples expected in clinical trials. All study procedures were conducted in accordance with the Declaration of Helsinki.

**Table 1:** Patient Demographics and Tumor Characteristics

	All Patients (N = 40)
Sex, n (%)	
Men	14 (35)
Women	20 (50)
Missing	6 (15)
Median age,* yrs (range)	67 (35-94)
	
Primary diagnosis, n (%)	
Colon adenocarcinoma	36 (90)
Rectal adenocarcinoma	3 (7.5)
Rectal carcinoma	1 (2.5)

*Age data were not available for 7 patients.

Mutational analysis of *KRAS *sequences was performed by the Amgen DNA Sequencing Laboratory and 5 independent laboratories that provide diagnostic services for academic/clinical research laboratories and/or clinical trials. Each laboratory was provided with 10-μm tissue sections from all 40 samples in 2007 except Agencourt. Agencourt was provided with extracted genomic DNA from the remaining 35 specimens in 2009. DNA was extracted using the QIAamp FFPE Tissue kit (Qiagen Inc, Carlsbad, CA) according to the manufacturer's instructions, with the addition of a 16-hour proteinase K lysis step.

### Direct Sequencing of KRAS by the Amgen DNA Sequencing Laboratory

Exon 2 of *KRAS *was amplified from isolated genomic DNA using the Roche Expand Long Template PCR System (Roche Applied Science, Indianapolis, IN). The forward primer sequence was 5'-AAGGTACTGGTGGAGTATTTG-3', and the reverse was 5'-GTACTCATGAAAATGGTCAGAG-3', resulting in a predicted amplicon length of 295 bp. Cycling conditions were as follows: 93°C, 3 minutes; 40-46 cycles at 93°C, 15 seconds; 62°C, 30 seconds; 72°C, 30 seconds; and 72°C, 4 minutes. Polymerase chain reaction (PCR) products were directly sequenced in triplicate (3730xl DNA Analyzers; Applied Biosystems). Sequences were analyzed using Sequencher^® ^software (Gene Codes Corporation, Ann Arbor, MI). Results were reported when all 3 parallel PCR products generated 4 acceptable sequences, resulting in a total of 12 sequences for each sample.

### Commercial KRAS Mutation Analysis

Five commercial services were contracted to analyze *KRAS *mutational status. HistoGeneX (Antwerp, Belgium) used the DxS K-RAS Mutation Test Kit (DxS Ltd, Manchester, UK) that interrogates the 7 most common somatic mutations of codons 12 and 13 (G12A, G12D, G12R, G12V, G12C, G12S, and G13D) using allele-specific PCR amplification with an amplification-refractory mutation system technology in combination with Scorpion^® ^detection to measure amplification products by real-time PCR [5,6]. The DxS K-RAS Mutation Test Kit is capable of detecting approximately 1% of mutated *KRAS *from background wild-type DNA.

Gentris Clinical Genetics, Inc. (Morrisville, NC, USA) assessed *KRAS *mutational status by direct DNA sequencing of codons 12 and 13. Gentris used a pathology service (Integrated Laboratory Systems, Inc.) to enrich for tumor tissue by removing normal tissue from the slides and then extracting genomic DNA from the remaining material. Isolated genomic DNA was then amplified with primers that spanned codons 12 and 13 of *KRAS*, and the resulting PCR products were subjected to cycle sequencing in reverse reactions.

Genzyme Corporation (Cambridge, MA) used an allele-specific primer extension technique and reported results for any mutation of codons 12 and 13.

Invitek GmbH (Berlin, Germany) used their Invisorb^® ^Spin Tissue Mini Kit for DNA extraction and evaluated *KRAS *mutational status using their Invigene^® ^K-ras Genotyping Kit, which employs allele-specific oligonucleotide hybridization. Results were reported for 6 possible mutations at each of codons 12 and 13.

Agencourt Bioscience Corporation conducted picotiter plate pyrosequencing using the Roche 454 system described previously [7-9]. Briefly, unique barcode-containing primers were used to amplify *KRAS *exon 2. All the uniquely tagged samples were pooled at equimolar concentrations and adaptors were ligated to allow bead binding. Optimized mixing conditions were then used to ensure that each bead-bound DNA molecule was within an oil droplet that contained PCR reagents. Emulsion PCR was performed using primers that included biotin to enable selective capture of those beads where successful amplification took place. The emulsion was subsequently broken, the biotin-containing beads captured, and each bead loaded by centrifugation into 1 well of a picotiter plate. Traditional pyrosequencing was performed, yielding a library of sequences linked to unique barcodes. Results were reported as the sequences derived from flowgrams, together with the 10-bp sequences employed for each sample to allow deconvolution of the data at Amgen. The number of mutant and wild-type sequences for each sample was reported.

### Statistical Analysis

*KRAS *genotype was classified as either wild type or mutant. Techniques were considered to be in agreement if both identified wild type or a mutant, even if the allele reported was different. Agreement was assessed by κ statistics [10] using SAS/STAT^® ^software version 9.1 (SAS Institute, Cary, NC).

## Results

### Results of Direct Sequencing by the Amgen DNA Sequencing Laboratory

Twenty (50%) patients had *KRAS *exon 2 mutations in sequencing performed by Amgen: G13D (n = 10), G12D (n = 5), G12V (n = 5), G12A (n = 2), and G12S (n = 1) (Table 2). Three of the G13D mutations occurred in combination with a codon 12 mutation.

**Table 2:** *KRAS *Mutations Reported by Mutation Analysis Method

Sample	Direct Sequencing (Amgen)	Allele-Specific PCR (HistoGeneX/DxS)	Direct Sequencing (Gentris)	Allele-Specific Hybridization (Invitek)	Allele-Specific PCR Extensions (Genzyme)	Picotiter Plate Sequencing (Agencourt)*
Samples for which a *KRAS *sequence determination was made, n	40	40	32	27	35	32
Total WT, n	20	21	18	3	15	15
1	G13D	G13D	G13D	G13D	G13D	G13D (4219/7836; 0.54)**
2	WT	WT	WT	Inconclusive^†^	WT	WT (8880)**
3	G13D	G13D	12WT, no call 13^†^	G13D	G13D	G13D (1679/8282; 0.2)
4	WT	WT	WT	Inconclusive^†^	WT	WT (8371)
5	WT	WT	WT	G13D	WT	Not tested
6	G12A	G12A	G12A	WT	G12A	No result^†^
7	G12S, G13D	G12S	G12S, no call 13^†^	Inconclusive^†^	Failed^†^	G12S (53/825; 0.06)
8	G12V, G13D	G12V	G12V	G12S, G12V	G12V	G12V (244/1289; 0.19), G13D (53/1289; 0.04)
9	WT	WT	WT	Not analyzed^‡^	Failed^†^	G12V (43/106; 0.41)
10	WT	WT	12WT, no call 13^†^	WT	Failed^†^	WT (18)
11	WT	WT	Mixed sequence^§^	Not analyzed^‡^	Failed^†^	No result^†^
12	G12V	G12V	G12V	G12D, G13D	G12V	G12V (2443/4707; 0.52)
13	WT	WT	WT	G13D	WT	WT (9223)
14	WT	WT	WT	Inconclusive^†^	WT	WT (9921)
15	G12D	G12D	G12D	G12D, G13D	G12D	G12D (4178/8006; 0.52)
16	WT	WT	WT	Inconclusive^†^	WT	WT (7001)
17	G13D	G13D	No call 12 or 13^†^	G13D	G13D	Not tested^||^
18	G12A	G12A	G12A	G12A, G13D	G12A	Not tested^||^
19	WT	WT	WT	G13D	WT	WT (8891)
20	G12D	G12D	G12D	G12D	G12D	G12D (2513/4304; 0.58)
21	G13D	G12D	WT	G12D, G13D	G12D, G13D	G12D (761/6260; 0.12), G13D (1639/6260; 0.26)
22	WT	WT	WT	Inconclusive^†^	WT	WT (2908)
23	G12D	G12D	WT	Inconclusive^†^	G12D	G12D (2115/7262; 0.29)
24	WT	WT	No amplification^¶^	WT	Failed^†^	WT (71)
25	WT	WT	WT	G13D	WT	WT (2477)
26	WT	WT	WT	Inconclusive^†^	WT	WT (5887)
27	G12V	G12V	G12V	G12V	G12V	G12V (1812/6404; 0.28)
28	WT	WT	WT	Inconclusive^†^	WT	WT (8696)
29	WT	WT	G12V	G13D	WT	WT (3876)
30	G13D	G13D	G13D	G13D	G13D	G13D (4697/9754; 0.48)
31	WT	WT	No amplification	G12D, G13D	G13D	No result^†^
32	WT	WT	WT	G13D	WT	WT (5048)
33	G12V	G12V	WT	G12V, G13D	G12V	G12V (505/1042; 0.48)
34	WT	WT	WT	G13D	WT	WT (6329)
35	G13D	G13D	G13D	G13D	G13D	G13D (2578/7158; 0.36)
36	G12V, G13D	G12V	G12V	Inconclusive^†^	G12V	Not tested^||^
37	G12D	G12D	G12D	G12D, G13D	G12D	G12D (14/61; 0.23)
38	G13D	WT	12WT, no call 13^†^	G13D	G13D	G13D (1539/5423; 0.28)
39	WT	WT	WT	G13D	WT	Not tested^||^
40	G12D	G12D	G12D	Inconclusive^†^	G12D	G12D (1884/4565; 0.41)

PCR = polymerase chain reaction; WT = wild type.

*Numbers in parentheses indicate the proportion of sample DNA containing the mutant allele.

^†^Service was unable to conclusively determine genotype.

^‡^Sample was determined to be unsuitable for subsequent hybridization.

^§^Multiple observed sequences precluded deconvolution of sequence traces.

^||^Samples no longer available for testing.

^¶^*KRAS *sequence could not be amplified by PCR.

**The single number in parentheses refers to the total sequence reads for that sample when all were WT, and for MT, the fraction reflects the number of sequence reads for a given mutation over the total number of sequence reads for that sample, followed by the numerical fraction of that ratio.

### Comparability of KRAS Mutational Analysis

Variation was observed in the comparability of direct sequencing by Amgen and the results of commercial analyses as shown by the tabulation of the reported results (Table 2) and the comparison of these results using the κ statistic (Table 3). The results of allele-specific testing by HistoGeneX using the DxS kit (κ = 0.95; 95% CI, 0.83—1.00) and by Genzyme (κ = 0.94; 95% CI, 0.85—1.00) closely matched the results of direct sequencing by Amgen, differing on 2/40 samples and 1/35 samples, respectively. The results of picotiter plate sequencing by Agencourt were also almost in perfect agreement with the results of direct sequencing by Amgen (κ = 0.94; 95% CI 0.52—0.98; 32 results reported from 35 samples). Results from the 2 techniques differed in only 1 sample when making a determination of *KRAS *wild-type versus mutant sequence. Amgen direct sequencing identified sample 9 as wild type, whereas Agencourt picotiter plate sequencing detected a G12V mutation. The results of direct sequencing by Gentris were in substantial agreement with the results of direct sequencing by Amgen, with disagreement on 4 samples (κ = 0.75; 95% CI, 0.52—0.98; 32 results reported from 40 samples). The results of mutational analysis by allele-specific oligonucleotide hybridization by Invitek (as conducted in 2007) were in slight agreement with the results of direct sequencing by Amgen (κ = 0.13; 95% CI -0.15—0.42; 27 results reported from 40 samples). *KRAS *analysis by Invitek identified only 3 wild-type samples, 2 of which were in agreement with direct sequencing by Amgen.

**Table 3:** Comparability of *KRAS *Mutational Analysis Using the κ Statistic

κ (95% CI) [n]*	Allele-Specific PCR (HistoGeneX/DxS)	Direct Sequencing (Gentris)	Allele-Specific Hybridization (Invitek)	Allele-Specific PCR Extensions (Genzyme)	Picotiter Plate Sequencing (Agencourt)
Direct sequencing (Amgen)	0.95 (0.85, 1.00) [40]	0.75 (0.52, 0.98) [32]	0.13 (–0.15, 0.42) [27]	0.94 (0.83, 1.00) [35]	0.94 (0.82, 1.00) [32]
Allele-specific PCR (HistoGeneX/DxS)		0.75 (0.52, 0.98) [32]	0.11 (–0.16, 0.37) [27]	0.89 (0.73, 1.00) [35]	0.88 (0.71, 1.00) [32]
Direct sequencing (Gentris)	0.75 (0.52, 0.98) [32]		–0.09 (–0.27, 0.08) [21]	0.74 (0.51, 0.98) [31]	0.63 (0.35, 0.92) [27]
Allele-specific hybridization (Invitek)	0.11 (–0.16, 0.37) [27]	–0.09(–0.27, 0.08) [21]		–0.08(–0.22, 0.06) [25]	0.29 (–0.05, 0.63) [21]
Allele-specific PCR extensions (Genzyme)	0.89 (0.73, 1.00) [35]	0.74 (0.51, 0.98) [31]	–0.08(–0.22, 0.06) [25]		1.00 (1.00, 1.00) [28]
Picotiter plate sequencing (Agencourt)	0.88 (0.71, 1.00) [32]	0.63 (0.35, 0.92) [27]	0.29 (–0.05, 0.63) [21]	1.00 (1.00, 1.00) [28]	

PCR = polymerase chain reaction.

*The κ statistic [10] measures agreement for *KRAS *mutational status (wild type vs mutant), with a value of 1.0 indicating perfect agreement and a value of -1.0 indicating perfect disagreement. Values in brackets are the 95% CI for the κ statistic. Values in square brackets indicate the number of samples being compared by the κ statistic (ie, the number of samples for which the 2 assays being compared both had a determination of wild-type or mutant).

The quantitative nature of the Agencourt picotiter plate sequencing assay, which generates counts of mutant and wild-type sequences, provided additional data that, in many instances, allowed clarification of the results from other laboratories (Table 2). For example, 3 of the 5 other laboratories detected the G12S mutation in sample 7, whereas picotiter plate sequencing identified the mutant allele in 6% of the DNA. In sample 21, direct sequencing by Amgen identified a G13D mutation, HistoGeneX detected a G12D mutation, and Invitek and Genzyme detected both G13D and G12D mutants. Picotiter plate sequencing identified G13D and G12D mutations in 26% and 12% of the DNA, respectively. In sample 9, picotiter plate sequencing identified a G12V mutation in 41% of sample DNA, whereas the other assays identified a wild-type genotype. It is unclear whether this is due to tumor heterogeneity or other factors.

## Discussion

The results of this study suggest that analyses of *KRAS *mutational status by most commercial services are accurate and are generally in close agreement with results of direct sequencing by Amgen. The results from Agencourt, HistoGeneX/DxS, and Genzyme were in almost perfect agreement with Amgen sequencing data and the results from Gentris were in substantial agreement with the Amgen analysis, whereas the results from Invitek were in slight agreement. It should be noted that Invitek has released a new assay since these comparisons were performed in 2007. The allele-specific oligonucleotide hybridization assay used in this study is no longer commercially available and has been replaced by an alternative technique. The frequency of *KRAS *mutation (20/40 as assessed by the Amgen laboratory) in the tumor biopsy specimens used in this study is consistent with that previously observed in clinical studies investigating *KRAS *mutations in CRC [11,12]. In 2007, these data (together with other considerations) led to the selection of the DxS assay performed by HistoGeneX for the evaluation of *KRAS *mutational status in samples from the panitumumab pivotal trial [4].

Several factors may have contributed to the lack of agreement between *KRAS *testing assays observed in this study. These factors may have included differences in the sensitivity and specificity of *KRAS *tests, differences in sample processing and DNA enrichment techniques between laboratories, and variation in tumor content between tumor samples and within tumor samples. Although direct sequencing is typically considered the gold standard for mutational analysis, emulsion PCR followed by picotiter plate sequencing may offer several advantages [13]. Because of the high sensitivity of picotiter plate sequencing, accurate detection of very low abundance mutations in a tumor sample is feasible [7]. Indeed, this highlights an important issue regarding sample selection for concordance studies of somatic mutation analysis in solid tumor specimens. Ideally, samples for concordance studies would comprise serial sections from a single homogeneous tumor block, allowing both preanalytical and analytical variables to be measured as a composite from a single sample. However, serial sections from a single block distributed amongst analytical platforms is not ideal given the likelihood of variable ratios of tumor:normal:stromal:necrotic tissue among sections and the potential for tumor heterogeneity (as suggested by results for samples 8 and 21, which contained 2 different mutations that might vary in ratio through the block). We propose that concordance testing should be a 2-step process using separate sets of samples. The first would determine the ability to successfully extract PCR-amplifiable DNA (by employing sections from a range of tumor blocks with variable tumor:normal:stromal:necrotic tissue ratios) with a second set of samples (comprising aliquots from DNA extracts with predetermined mutant:wild-type somatic mutation ratios) to determine the ability to correctly report mutations. Some of these samples should be close to the lower level of sensitivity expected of such assays (eg., 1%).

*KRAS *mutation assays may provide physicians with guidance when making decisions on the use of anti-EGFR therapy in the treatment of metastatic CRC [14]. However, the lack of concordance among results of some assay techniques emphasizes the importance of a rigorous quality assurance program to ensure that results are of high quality [15-17], potentially using the sample types described above.

Whitehall et al recently published a study comparing 6 *KRAS *mutation assays in the analysis of primary colorectal cancer samples [18]. As in this study, most techniques assessed were found to be in good agreement, with some notable exceptions. The key strength of our study was that laboratories (with the exception of Agencourt) analyzed *KRAS *from tumor sections on glass slides rather than from DNA, and the results were consequently a closer mimic of clinical testing procedures. A recent study by Weichert et al compared 4 *KRAS *mutation assays of primary colorectal cancer samples and metastases using DNA extracted from tumor sections on glass slides and similarly determined that the assay techniques were generally in good agreement [19].

## Conclusions

The results of this study suggest that most (but not all) commercial services provide analysis that is accurate and comparable with direct sequencing. However, the lack of concordance among results of some assay techniques, suggest that rigorous quality assurance programs will be necessary to ensure consistent and accurate results.

## Competing interests

KO, TJ, SS, MW, IS, DJF, TG, WB, AB, AP, and SDP are employees and shareholders in Amgen Inc.

## Authors' contributions

KO, TJ, SS, IS, DJF, and SDP contributed to the conception and design of the study; IS, TG, and WB provided study material; TJ, SS, TG, WB, AB, and AP provided study materials; TJ, MW, and AP analyzed and interpreted the data; KO and SDP wrote the manuscript. All authors read and approved the final manuscript.
